# Defining gentrification for epidemiologic research: A systematic review

**DOI:** 10.1371/journal.pone.0233361

**Published:** 2020-05-21

**Authors:** Nrupen A. Bhavsar, Manish Kumar, Laura Richman

**Affiliations:** 1 Department of Medicine, Duke University School of Medicine, Durham, North Carolina, United States of America; 2 Trinity School of Arts and Sciences, Duke University, Durham, North Carolina, United States of America; 3 Department of Population Health Sciences, Duke University School of Medicine, Durham, North Carolina, United States of America; University of the Witwatersrand, SOUTH AFRICA

## Abstract

Neighborhoods have a profound impact on individual health. There is growing interest in the role of dynamic changes to neighborhoods–including gentrification–on the health of residents. However, research on the association between gentrification and health is limited, partly due to the numerous definitions used to define gentrification. This article presents a systematic review of the current state of literature describing the association between gentrification and health. In addition, it provides a novel framework for addressing important next steps in this research. A total of 1393 unique articles were identified, 122 abstracts were reviewed, and 36 articles published from 2007–2020 were included. Of the 36 articles, 9 were qualitative, 24 were quantitative, and 3 were review papers. There was no universally accepted definition of gentrification; definitions often used socioeconomic variables describing demographics, housing, education, and income. Health outcomes associated with gentrification included self-reported health, preterm birth, mental health conditions, alcohol use, psychosocial factors, and health care utilization, though the direction of this association varied. The results of this review also suggest that the impact of gentrification on health is not uniform across populations. For example, marginalized populations, such as Black residents and the elderly, were impacted more than White and younger residents. In addition, we identified multiples gaps in the research, including the need for a conceptual model, future mechanistic studies, and interventions.

## Introduction

Neighborhoods are an integral component of the social determinants of health, with a substantial body of literature reporting associations between the neighborhood in which an individual lives and their risk for multiple health conditions. Prior research suggests a positive, linear relationship between neighborhood wealth and better health outcomes [1], with residents of wealthier neighborhoods generally receiving the health benefits of greater access to green space [2], healthier food options [3], less crime [4], and other amenities, as compared to residents of poorer neighborhoods. Increasingly, researchers have been interested in understanding the population health impacts of changes in neighborhoods over time [5].

As population trends indicate that the neighborhoods of major cities are experiencing rapid demographic and socioeconomic shifts [6], there is a growing need to understand the differential impact that these changes have on neighborhood residents and community dynamics. One such change occurs when a neighborhood becomes upwardly mobile through an influx of younger and/or higher SES individuals, resulting in increased housing prices and a higher cost of living—a process generally referred to as “gentrification [7, 8].”

Gentrification has been examined extensively in economics and sociology literature, broadly encompassing themes around the influx of higher-income households, changes to the racial composition of neighborhoods, and the displacement of lower-income individuals [9–11]. Outcomes have focused on the impact that gentrification has on crime, labor markets, and education opportunities. Our analysis focuses specifically on what is arguably downstream of these other outcomes: the impact of gentrification on health. As epidemiological research increasingly studies the impact of social determinants of health, it is critical to examine both the ways in which processes such as gentrification are defined and how a given definition may affect its association with the health of neighborhoods and the individuals residing within them.

Researchers have not reached a consensus as to whether gentrification has a net positive or negative impact on the health of communities, largely due to variation in the literature regarding both how gentrification is defined and the wide range of outcomes examined. To date, there has been minimal effort to forge a consensus on the optimal definition of gentrification. This lack of a common definition has further stymied a comprehensive understanding of gentrification’s potential health impacts on residents of changing neighborhoods. This systematic review aims to fill that gap, critically evaluating research that has examined relationships between gentrification, health, and determinants of health, while also identifying the quantitative definitions used by articles to phenotype gentrification. We summarize studies that look at different proximal and distal health outcomes related to gentrification. Based on our comprehensive review of this literature, we also propose a conceptual model to guide future research on gentrification and health.

## Methods

A recent rule change (as of October 2019) prevents reviews that have begun the process of data extraction from registering their protocols on the International Prospective Register of Systematic Reviews (PROSPERO). We were, therefore, unable to register our protocol. However, both our protocol and review, described below, follow the 2009 PRISMA Statement [12], as outlined in the supporting information (S1 Table).

### Search strategy

Systematic searches of the literature were conducted using PubMed and Web of Science to identify articles published through March 12, 2020. Our initial search used a sensitive term (i.e., “gentrification”), allowing us to understand the extent of literature on this subject. This was followed by a more specific search using keywords related to three main concepts: gentrification, health, and mechanisms and moderators through which gentrification may affect health.

Gentrification was conceptualized broadly, incorporating terms related to the process, such as “urban renewal,” “urbanization,” “displacement” and “eviction.” The terms “health” and “social determinants” were used to capture articles measuring health outcomes. Lastly, to include articles discussing the mechanisms and moderators that may affect the relationship between gentrification and health, our search included terms such as “inequity,” “equity,” “social capital,” “race”, and “socioeconomic status.” A complete list of terms used in in our search can be found in the supporting information (S1 Appendix).

Literature searches were rerun every week to ensure inclusion of relevant and newly published articles. References cited by included articles were also screened for relevance. In addition, all original studies from included reviews were examined for inclusion into our review.

### Inclusion and exclusion criteria

Articles included in this review focus on the relationship between gentrification and constructs of health. Peer-reviewed qualitative, quantitative, and review articles, both within and outside the United States, were considered for inclusion. This review aims to identify the phenotypes used to define gentrification, and therefore, did not formally define gentrification a priori; we included articles examining the process in relation to its impact on health. Although associated terms such as “urban renewal” and “urbanization” were included in the literature search, articles using these terms were ultimately excluded, as these terms were too broad and/or did not adequately capture the process of gentrification. Additional exclusion criteria included: non-English language, not peer-reviewed, health not measured as a dependent variable, and no explicit definition of gentrification.

The dependent variable of interest was health and was broadly defined. We focused on one or more of the following health measures and outcomes: self-reported health, sub-clinical conditions (e.g., hypertension, diabetes), health-related behaviors (e.g., alcohol consumption), environmental changes that impact health (e.g., park construction), and social determinants of health (e.g., housing insecurity) with implications for health. Peer-reviewed literature examining the mechanisms by which gentrification may affect health were also included.

### Study selection

**Fig 1:**
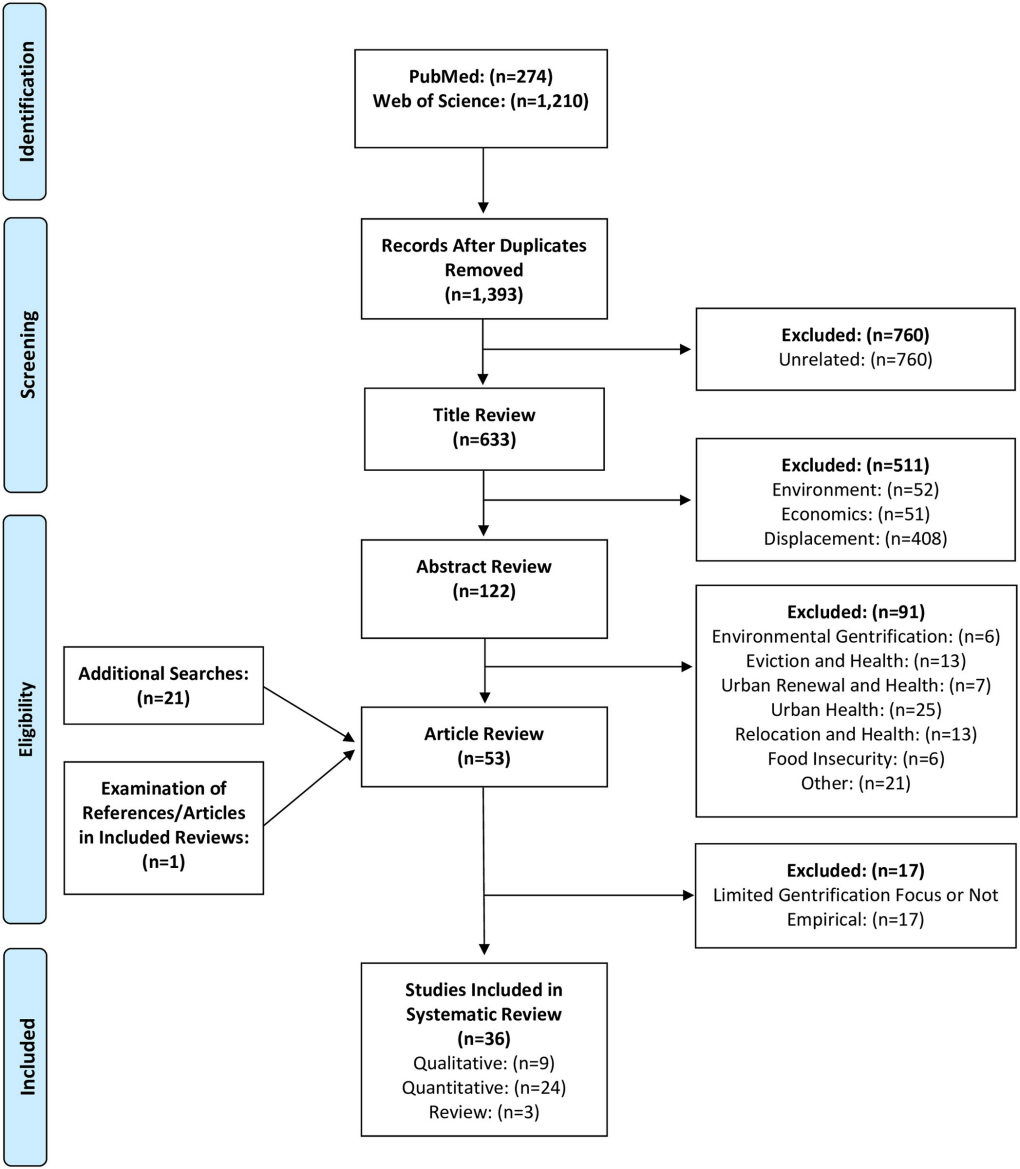
PRISMA flowchart.

The initial searches yielded 1,484 articles. After removing duplicates (n = 91), articles with unrelated titles (n = 760), and those that did not discuss gentrification or health specifically (n = 511), 122 articles were selected for abstract review. After eliminating articles that did not meet inclusion criteria, 31 articles were selected for a full review. Of these, 21 met the inclusion criteria. Repeated searches following the initial review date and utilizing the same screening process yielded 21 additional articles for full review and 14 for inclusion, for a sum of 35 peer-reviewed articles (Fig 1). One additional article was found by reviewing articles included in systematic reviews and references cited within original research. This resulted in a total of 36 peer reviewed articles.

All three co-authors participated in the full text review and discussed all articles that were included in this manuscript. Potential disagreements were resolved with a majority consensus on the study’s final inclusion.

### Data extraction

For data extraction, included articles were categorized into qualitative, quantitative, and review articles. The following study characteristics were extracted and recorded onto a pre-defined data abstraction form: author(s), title, manuscript type, study population, variables used to define gentrification, definition of gentrification, sources of data, analytical methods, and a summary of article findings. Additionally, all articles were organized into one or more of the following health categories: self-reported health, health related behaviors, environmental changes that have implications for health or health behaviors, moderators/group differences, potential mechanisms (social capital and psychosocial stress), and health outcomes.

For articles quantitatively defining gentrification, we summarized the variables used to define gentrification, geographic level used to identify gentrification, and the time between the baseline and follow-up assessment to measure neighborhood change.

### Quality assessment

To assess the quality of studies included in the review we used three tools. For quantitative studies, we used an assessment tool developed jointly by the National Institute of Health and Research Triangle Institute International [13]. For qualitative studies, we used an assessment tool developed by the National Institute for Health and Care Excellence [14]. For systematic reviews, we used the guidelines of the respective protocols the reviews adhered to (PRISMA and RAMESES) [12, 15]. All three co-authors reviewed the studies and dual review was conducted on 20% of the studies. Disagreements between two co-authors reviewing a study were adjudicated by the third co-author.

## Results

### Overview

The literature search and subsequent full text review yielded 36 eligible papers, which were published from 2007 to 2020 (Fig 1). Detailed information on the main characteristics of quantitative articles can be found in Table 1a, qualitative articles in Table 1b, and reviews in Table 1c. Most studies were conducted in the United States [16–40]. A handful of studies were conducted in Spain, Australia, and Canada [5, 28, 41–49]. Of the 36 papers included in this literature review, 6 were qualitative, 24 were quantitative, and 3 were review papers. Multiple health outcomes were not associated with gentrification [19, 21, 23–25, 30, 36, 37, 48, 49]. Health outcomes that were associated with gentrification included: self-reported health [18, 21, 22, 25, 32, 44], preterm birth [23], mental health conditions [19, 32, 38, 44], alcohol and drug use [24, 27, 43, 47], psychosocial factors [17, 20, 28, 31, 33, 40, 42, 46, 49], and health care utilization [26].

**Table 1:** **a**. Descriptive summary of included quantitative articles. **b**. descriptive summary of included qualitative articles. **c**. Descriptive summary of included review articles.

**A**						
**Citation**	**Definition/Method of Gentrification**	**Source of Gentrification Data**	**Health/ Health Related Outcome**	**Summary of Findings**	**Study Location**	**Analytical Methods**
Gibbons, J. et al. (2018)	**Eligibility**: MHI below reference city, 1990–2000	CDC 500 Cities Project, 1990 Census, 2000 Census, 2010–2014 ACS	Self-reported health	Gentrification associated with higher SRH at neighborhood level, no effect on city-wide level	United States	Multilevel models and ordinary least squares (OLS) model
	**Gentrified**: increase in gross rent or MHI above reference city median and increase in college-educated residents above reference city median (1990–2000, 2000–2014)					
Gibbons, J. et al. (2016)	**Eligibility**: MHI below city wide median	2008 Philadelphia Health Management Corporations Southeastern Pennsylvania Household Health Survey, 2006–2010 ACS, 2000 Census	Self-reported health and Moderators/Group Differences	Gentrification had a marginal effect improving overall SRH, but blacks in gentrifying tracts were more likely to report worse SRH. Accounting for racial change, white gentrification had no measurable effect on minority health, though black gentrification led to worse SRH for blacks.	Philadelphia, United States	Multilevel logistic regression model
	**Gentrified**: Increase in gross rent or MHI above the citywide median and increase in college-educated residents above the citywide median.					
	White gentrification was gentrifying tracts experiencing an increase in white residents.					
	Black gentrification was gentrifying tracts experiencing an increase in black residents					
Izenberg, J. et al. (2018)	**Eligibility: MHI below** median for CBSA, prop. building stock pre-dating 1980 exceeded that of median tract for CBSA, at least 50% of the census block groups “urbanized” according to 2010 Office of Management and Budget guidelines.	California Health Interview Survey, 2006–2010, 2011–2015 ACS	Self-reported health and Moderators/Group Differences	Gentrification was not associated with a SRH overall. However, among black residents, gentrification accounted for a 144% increase in the odds of fair/poor SRH.	California, United States	Multivariable logistic regression models
	**Gentrified**: Tract level increase in 2015-adjusted median rent and education attainment.					
Smith, RJ et al. (2018)	**Eligibility**: Neighborhood MHI less than 40th percentile of the metropolitan area and resided in a primary city or inner-ring suburb	National Health & Aging Trends Study, 1970–2010 National Neighborhood Change Database.	Self-reported health, depression/anxiety symptoms and Moderators/Group Differences	Economically vulnerable older adults in gentrifying neighborhoods had higher SRH than those in low-income neighborhoods and more depression and anxiety symptoms than those in affluent areas. Higher-income older adults in gentrifying neighborhoods had poorer mental health than those in low-income neighborhoods and more depression and anxiety symptoms than those in affluent areas	United States	Matching design and linear regression
	**Gentrified**: Increase in MHI, percent college-educated residents, median owner-occupied housing value, median rent.					
Cole, HWS., et al. (2019)	**Eligibility**: Identified zip codes and ZCTAs where more than 50% of residents fell into low-income category, based on 2000 census data.	New York City Department of Health and Mental Hygiene Community Health Survey; New York City Department of Parks and Recreation 2000 American Census 2006–2010 ACS	Self-reported health and access to "active" green spaces (walkways, greenways, parks, etc.)	Greater exposure to active green space associated with lower odds of fair or poor SRH. Residents in gentrifying areas seem to benefit from increased access to green space. Within gentrifying areas, only those with high incomes or high education benefitted from increased green space.	New York City, United States	Logistic regression modeling
	**Gentrified**: Calculated a gentrification score comparing rates of change for 7 indicators within zip code areas to city-wide. If rate of change was greater for the zip code than for the city for > = 4 indicators, neighborhood was considered gentrifying. For indicators representing changes in socially vulnerable populations, coding was opposite to be considered a sign of gentrification					
Lim S, et al. (2017)	**Eligibility**: Ranked neighborhoods by 3 characteristics in 2005 and by linear growth on each characteristic during 2005–2014	2006–2014 ACS, 2006–2014 Statewide Planning and Research Cooperative System	Health Related Behaviors (ED visits and hospitalizations due to mental health)	Compared to those who remained in gentrifying neighborhoods and residents in non-gentrifying neighborhoods, displaced residents were more likely to make emergency department, experience hospitalizations, and make mental health visits.	New York City, United States	Principal Component Analysis (PCA) and logistic regression analysis
	**Gentrified**: Based on 6 rankings (in 2005 and from 2005–2014), gentrifying neighborhoods had low initial ranking and rapid increases in 3 variables. Used PCA to identify neighborhoods meeting definitions.					
Izenberg, J. M., et al. (2018)	**Eligibility: MHI below** median for CBSA, prop. building stock pre-dating 1980 exceeded that of median tract for CBSA, at least 50% of the census block groups “urbanized” according to 2010 Office of Management and Budget guidelines.	California Health Interview Survey, 2006–2010, 2011–2015 ACS	Health Related Behaviors (binge drinking)	Overall, gentrification was not associated with binge drinking. Gentrification was significantly associated with binge drinking for community members who had resided in their neighborhood for less than 5 years.	California, United States	Multivariable logistic regression models
	**Gentrified**: Tract level increase in 2015-adjusted median rent and education attainment.					
Gullon P, et al. (2017)	**Eligibility**: Increase in the percent of residents with high education from 2005–2014	2005 and 2014 Census data for the city of Madrid, Padrón Spain, Social Security and Employment Service Registry, Idealista Report	Environmental Changes and Implication for Health (Changes to neighborhood walkability)	Higher SES neighborhoods had less walkability. This was relationship was moderated by gentrification.	Madrid, Spain	Mixed linear models
	**Gentrified**: Neighborhoods as those in top 95% of rank change.					
Abel, T et al. (2011)	**Eligibility**: None listed	US EPA Risk Screening Environmental Indicators (RSEI), Neighborhood Change Database, 1990 Census and 2000 Census.	Environmental Changes and Implication for Health (toxic air exposure)	Clusters 13 and 15, which experienced the least gentrification, accounted for 68% of cumulative toxic air pollutants risk between 1990 and 2007. Higher prop. of minority and working class residents were concentrated in neighborhoods near Seattle's worst industrial pollution risk.	Seattle, United States	Principal Component Analysis (PCA) and cluster analysis using Ward's Method
	**Gentrified**: Based on 12 socioeconomic variables—used principal component analysis and cluster analysis to group neighborhoods that gentrified					
Anguelovski, I. et al. (2018)	**Eligibility**: None listed	Barcelona Statistics Department, Barcelona Municipal Department of Fiscal Studies, Barcelona Parks and Gardens Institute, Census Data from 1991, 1996, 2001, and 2004–2006	Environmental Changes and Implication for Health (access to green space)	“Green gentrification” observed in several socially vulnerable neighborhoods. MHI, percentage of the population with bachelor's degrees, immigration of people from the Global North were greater near several of the parks. relative to the district overall. Some areas experienced lower relative change in percent 65+.	Barcelona, Spain	Changes in housing and population trends and local and global regression techniques
	**Gentrified**: Baseline was year park was created with Follow-up at most recent year w/ data available. Changes to MHI, prop. of residents 65+, prop. of residents with bachelor's degrees, and prop. of immigrants from Global South near parks was compared to district level changes.					
Linton SL, et al. (2017)	Gentrification measured by an index of % change from 1990–2009 in: % poverty, % college or more among adults > = 25, % White, MHI and median rent. Once confirmed through PCA, the items were standardized by z-score, weighted by factor loadings and summed to create the index.	1990 Census (Readjusted to 2010 Census Tract Boundaries), 2007–2011 ACS, 2009 HUD Picture of Housing	Health Related Behaviors (People who inject drugs)	There is a significant positive association between zip code level gentrification and homelessness among people who inject drugs.	United States	Univariate and multivariable multilevel logistic regression models
Huynh, M. and Maroko, A. (2014)	**Eligibility**: None listed	2005–2009 ACS, 2000 Census, New York City Department of Health and Mental Hygiene data 2008–2010	Moderators/Group Differences (preterm birth)	Gentrification not associated with pre-term birth overall. Very high gentrification was adversely associated with preterm birth for non-Hispanic Blacks. For non-Hispanic Whites, very high gentrification was protective in regard to preterm birth.	New York City, United States	Generalized estimating equation model
	**Gentrified**: Percent change and then z-scores were calculated for each of the three Census variables (MHI, college education level, and poverty level) and then summed. Lower z-scores corresponded to less gentrification. Quintiles marked stages of gentrification (very high, high, medium, low, and very low.					
Gibbons, J. (2019)	**Eligibility**: Median household income below that of the city in which they were located in 2000	2000 Decennial Census, 2006–2010 ACS, 2008 and 2010 waves of the Public Health Management Corporation’s (PHMC) Southeastern Pennsylvania Household Health Survey	Mechanisms of Health Implications (stress)	Gentrification marked by increases in whites and decreases in non-whites was significantly associated with above average stress in census tracts in Philadelphia	Philadelphia, United States	Multilevel logistic regression model
	**Gentrified**: Increase in gross rent or median home value above the citywide median and an increase in college-educated residents above the citywide median over 2000–2010					
Steinmetz-Wood M, et al. (2017)	**Eligibility**: Negative z-score in baseline year relative to Montreal census metropolitan area average and SD.	1996 Canadian Census, 2006 Canadian Census, ZEPSOM Study Wave One	Mechanisms for Health Implications (Social Capital)	Gentrification positively associated with collective efficacy. Those who moved into a gentrified neighborhood reported higher collective efficacy than those who lived in a non-gentrified neighborhood. Gentrification not linked to any physical or mental health outcomes.	Montreal, Canada	Multilevel linear regression
	**Gentrified**: Difference between baseline and follow-up (1996–2006) z-scores was positive for all indicators except for proportion of low income, which needed to be negative.					
Fong, P. et al.(2019)	**Eligibility**: None listed	Household Income and Labour Dynamics Survey Australia, MHI-5 of SF-36, Socioeconomic Index for Areas of Relative Advantage and Disadvantage (SEIFA, derived from Census data)	Self-reported Mental Health and Mechanisms and Implications for Health (Self-reported Neighborhood Identification)	Strong neighborhood identification acts as a buffer to protect individuals from mental health strains of gentrification.	Australia	Multilevel logistic regression model
	**Gentrified**: A positive value following the subtraction of the 2011 SEIFA index from the 2016 SEIFA index.					
Morenoff, J.D et al. (2007)	343 neighborhood clusters were measured for different indicators to create a set of factors in which to measure neighborhood context. All of the resulting factor scores were standardized to have a mean of zero and a standard deviation of one. First factor was socioeconomic disadvantage, second was characteristics belonging to neighborhood affluence and gentrification, third was ethnic/immigrant composition, and fourth factor as older age composition.	Chicago Community Adult Health Study (CCAHS), Project on Human Development in Chicago Neighborhoods (PHDCN), 2000 Census	Health Outcomes (blood pressure)	Blacks and people with lower levels of education have significantly higher odds of hypertension than their comparison groups (i.e., whites and people with 16 or more years of education), though this significance disappears when accounting for neighborhood context. In addition, neighborhood affluence and gentrification were associated with a lower risk of hypertension.	Chicago, United States	Multilevel logistic regression model
Dragan, K. et al. (2019)	**Eligibility**: MHI in the bottom 40% of city census tracts	New York State Medicaid Data, 2009–2017, ACS 2005–2009, 2011–2015	Health Related Behaviors, Health Outcomes (Overweight, Asthma, ADHD, Conduct disorder and anxiety or depression, ED visit and hospitalizations, Prop. of children with > = 1 well child visit (or routine exam)), Moderators/Group Differences	Gentrification was not associated with most outcomes. Diagnoses of anxiety or depression were significantly greater among children in rapidly gentrifying areas than those in persistently low SES areas. This difference was significant only for children who moved out of the neighborhood and those who stayed in market rate housing.	New York City, United States	Multivariable logistic regression models and multivariable liner regression models
	**Gentrified**: Growth in MHI through the study period and growth in the percent of college educated individuals placing census tracts in the top quarter of the neighborhoods; Moderately gentrifying if in the 11th to 25th percentile of growth and rapidly gentrifying if in the top 10th percentile of growth.					
Rhodes-Bratton, B. et al. (2018)	**Eligibility**: Categorization as a "low income" Sub Borough Area (SBA) in 1990, considered 0–80 Area Mean Income in 1990	Columbia Center for Children’s Environmental Health prospective birth cohort, National Time-Series Establishments, NYU Furman Center, NYC Department of Planning Community District Profiles	Environmental Changes and Implication for Health, Health Outcomes (food availability, BMI), and Moderators/Group Differences	Gentrifying neighborhoods experienced increases in healthy and unhealthy food chances. There was no relationship observed between gentrification and obesity.	New York City, United States	Linear and logistic regression models
	**Gentrified**: Neighborhoods that were considered low-income in 1990 and experienced rent growth above the median SBA rent growth between 1990 and 2014					
Sheringham, J. et al. (2017)	**Eligibility**: None listed	Index of Multiple Deprivation 2007, 2010, 2015, NHS General and Personal Medical Services workforce census, Episode Hospital Statistics	Health Related Behaviors (ED visits and hospitalizations due to mental health)	improvements in local equity performance, measured through relative falls in emergency admissions, were not a product of gentrification	England, United Kingdom	Linear regression model and administrative area level random and fixed effects regression models
	**Gentrified**: An extent of deprivation improving at least five places from the 2007 to the 2015 Index for Multiple Deprivation					
Bilal, U. et al. (2019)	A finite mixture model was used to categorize census tracts into one of four patterns of neighborhood change, with the use of 16 indicators: Declining SES, New Housing, Improving SES, and Stable Areas	EHR from primary care health centers in Madrid, Idealista Report, Servicio de Empleo Publico Estatal (National Employment Service), Padrón Spain, Cadaster	Health Outcomes (diabetes incidence)	Compared to those living in Stable areas, those living in Declining SES, New Housing and Improving SES areas have a decrease in diabetes incidence	Madrid, Spain	Finite mixture models
Schnake-Mahl, A. et al. (2020)	**Eligibility**: census tracts with an MHI in the bottom 40th percentile of the median county income.	1990 US Census, 2000 US Census, 2005–2009 ACS, Resilience in Survivors of Hurricane Katrina (RISK) project	Self-rated health and Health Outcomes (BMI and psychological distress)	There was no relationship between gentrification and health outcomes for participants in the RISK project who were displaced into gentrifying neighborhoods.	New Orleans, United States	Difference in difference models
	**Gentrified**: An index was created based off of changes to ratio of MHI in the census tract to MHI in the county. The baseline was the difference of the 2000 and 1990 ratio and was compared to that of 2005–2009 and 2000 ratio. “Gentrifying” tracts had a ratio from 2005–2009 > = 5 percentage points from the baseline.					
Narita, Z. et al., (2019)	**Eligibility**: None listed	The Survey of Police-Public Encounters II, Composite International Diagnostic Interview (CIDI), Neighborhood Change and Gentrification Scale (NCGS),	Health Outcomes (psychotic experiences)	Researchers found that there was no significant relationship between the occurrence of psychotic experiences and gentrification. Having a low income and racial minority status did not modify this association.	New York City and Baltimore, United States	Multivariable logistic regression models, a qualitative questionnaire was quantified to measure gentrification
	**Gentrified**: A composite measure was assigned using specific responses to the following questions on the NCGS. Higher composite scores using selected questions indicated greater gentrification					
Tran, L. et al. (2020)	**Eligibility**: census tracts had to be at or below 80% of their respective county's MHI at the start of the study period	2006–2010, 2011–2015 ACS, 2010 and 2015 Home Mortgage Disclosure Act (HMDA) aggregate reports, California Health Interview Survey (2011–2015)	Health Outcomes (serious psychological distress) and Moderators/Group Differences	This study found that living in a gentrified neighborhood was associated with an increased likelihood of serious psychological distress, as opposed to living in a low-income and not gentrified neighborhood. This negative impact on the mental health was seen in renters, low-income residents, and long-term residents, but not among homeowners, higher-income residents, and recent residents.	Southern California, United States	Probit models with instrumental variables
	**Gentrified**: PCA was conducted on 8 indicators of neighborhood change to develop neighborhood change categories. PCA scores were binned into groups and PCAs were stratified by county. Tracts in the group with the greatest PCA scores were considered "upscaled". Census tracts considered low-income (eligible to be gentrified) at the baseline that experienced "upscaling" were considered gentrified.					
Breyer and Voss-Andreae, (2013)	**Eligibility**: None listed	2000 US Census, 2010 US Census, 2006–2010 ACS, U.S. Census North American Industry Classification System (NAICS), dataset of SNAP retailers, Thrifty Food Plan (TFP)	Environmental Changes and Implication for Health (food availability)	This study found that food mirages (areas with a lack of access to affordable healthy food options) are most extreme in the gentrifying census tracts of Portland, though food deserts were not necessarily a problem.	Portland, United States	Stepwise linear regression; spatial lag regression
	**Gentrified**: gentrification was indicated by an increase in the percent of whites in a census tract.					
**B**						
Pennay, A., et al. (2014)	N/A—Districts described as “gentrified” have experienced a rapid increase in affluence and construction, though historically they have been home to working class and marginalized populations	Focus groups and qualitative interviews with street drinkers and social service providers	Health Related Behaviors (drinking)	Gentrification was largely seen as a cause of growing concerns over public drinking and the subsequent stereotyping and exclusion of street drinkers from public spaces. Some drinkers reported increased drinking and a loss of social connections following displacement.	Melbourne, Australia	Framework analysis
Whittle, J.H et al. (2015)	N/A—This article does not explicitly measure gentrification, but rather asses gentrification within the context of food insecurity among people living with HIV/AIDS in the San Francisco area.	UCSF, Project Open Hand; Recipients of food from Project Open Hand in San Francisco were recruited to participate in this study	Environmental Changes and Implication for Health (food availability)	Several participants linked food insecurity to the inability to pay for meals after having to pay for monthly rent. Authors surmise that increased rent prices, as a result of gentrification in the Bay area, exacerbate this situation.	San Francisco Bay Area, United States	Content analysis
Shmool, J. et al. (2015)	N/A—This article uses qualitative focus groups to determine neighborhood stressors in the five boroughs of New York City. Although gentrification is mentioned as a common stressor, it is not discretely measured.	15 focus groups in the five boroughs of New Yok City, three in each one. The median size of the focus group was 10, though the size ranged from 6–17.	Mechanisms of Health Implications (Psychosocial Stress)	The most common perceived stressors in the boroughs of New York City were perceived neglect and physical disorder, safety and harassment by police, and gentrification and racism. With gentrification, a common theme was the effect of gentrification on people of color, and the perceived racial preferences to the gentrifiers over those living in the original communities	New York City, United States	Constant comparative method and thematic analysis
Versey, H.S. (2018)	N/A—Central Harlem was chosen as the location for this study due to its "gentrifying" status, as described through large increases in median housing costs and median household income	Group Interviews of 9 senior housing sites in Central Harlem	Mechanisms for Health Implications (Social Capital) and Moderators/Group Differences	Common themes discussed included "newcomers changing things," and influx of white residents. In addition, participants reported a negative change in neighborhood trust and disruption of social networks as friends/family members moved out due to higher housing costs. A lack of collective efficacy and social spaces was also discussed.	New York City (Harlem), United States	Thematic analysis
Betancur, J. (2011)	N/A—Selects specific neighborhoods in Chicago and describes the process of gentrification in these neighborhoods (Uptown, Lake View, Lincoln Park, West Town, The Loop, Pilsen)	Qualitative, semi-structured Interviews	Mechanisms for Health Implications (Social Capital)	This paper mentions a source of conflict between gentrifiers and those affected by gentrification, with gentrifiers attributing the process to market forces and viewing it as beneficial to preserving communities' architecture and historical features. Those affected by gentrification often discussed it within the context of disrupting community and social fabric.	Chicago, United States	Exploratory analysis
Burns, V. F., et al. (2012)	Gentrification is defined as a phenomenon involving the “'invasion' of previously working-class neighborhoods by middle or upper-income groups and the subsequent displacement of many of the original residents"	30 Qualitative interviews of 30 residents (all older age) of the two neighborhoods studied.	Mechanisms for Health Implications (Social Capital) and Moderators/Group Differences	Gentrification may be associated with an increase in social exclusion, insecurity, and connectedness with the neighborhood. Gentrification was viewed as positive by some, though others described the process as leading to a loss in familiarity with their neighborhood.	Montreal, Canada	Inductive and deductive approaches to identify themes. Codes were generated using a grounded theory approach
Lyons, T., et al. (2017)	N/A—Gentrification was defined as "a global urban strategy tied to the development and accumulation of wealth that transforms neighborhoods to suit new residents"	33 Qualitative Interviews with trans sex workers	Mechanisms for Health Implications (Stress/Violence) and Moderators/Group Differences	Gentrification was often attributed to environmental and structural changes leading to the displacement of trans sex workers. Participants reported that their working conditions were increasingly unsafe because of overlapping structural vulnerabilities of construction activity, criminalization of sex work, and gentrification.	Vancouver, Canada	Thematic analysis and other "theory and data driven approaches"
Collins, A. et al. (2019)	N/A—Gentrification is defined as "the process of transforming vacant or low-income inner-city areas into economic, recreational, and residential use by middle-and upper-income individuals."	Qualitative, semi-structured Interviews with 72 people who use drugs in addition to 200 hours of ethnographic work	Mechanisms for Health Implications (Stress) and Health Related Behaviors	Policing practices in gentrifying areas present barriers to easy access of overdose prevention sites for people who use drugs, reinforcing their structural vulnerability. This may have downstream implications on mortality and health.	Vancouver, Canada	Thematic analysis
Versey, H.S. et al. (2019)	N/A—Central Harlem was identified as having undergone "gentrification" due to large increases in rental costs and home values. In addition, it was selected as the site for the study sue to its large but declining African American community.	Nine focus groups with 98 African American men and women living in the Central Harlem neighborhood. Neighborhood Change Survey by the NYU Furman Center (to provide evidence of gentrification)	Mechanisms for Health Implications (Social Capital)	Most participants felt a strong level of identification with their neighborhood and its people, one that was being taken away as newcomers moved into the area. This changing attitude was described by tensions participants had with "outsiders" and concerns about church tourism. Participants also reported financial pressures as a concern to aging in place and aging near family.	New York City, United States	Thematic analysis
**C**						
Mehdipanah R, et al. (2018)	N/A—this is a systematic review and they used "gentrification" as a search term so they didn't construct the variable and did not describe how identified studies constructed/defined gentrification.	Published papers	N/A	Suggests that gentrification may lead to increased levels of psychosocial stress for some members of the community, largely as a result of a disruption in social networks and community cohesion.	UK, Australia, US	This review was conducted according to RAMESES guidelines
Tulier, M.E et al. (2019)	Gentrification was defined as "a socio-economic process within neighborhoods where formerly declining disinvested neighborhoods experience reinvestment and in-migration of increasingly affluent new residents" for the systematic review but the definition of gentrification varied by study	Published papers	N/A	Recommends that studies offer a clear conceptualization of both gentrification and mechanisms studied, while considering both space and time.	United States	This review was conducted according to PRISMA guidelines
Schnake-Mahl, A. et al. (2020)	N/A—"Gentrification" was used as a search term and thus, no variable was constructed. In addition to gentrification, this review considered similar processes, such as urban regeneration, urban development, and neighborhood upgrading.	Published papers	N/A	This review suggests that the impact of gentrification on health varies across a variety of factors. Although most articles suggested that gentrification had a significant impact on health, the direction of this impact was unclear.	United States	This review was conducted according to PRISMA guidelines

SRH, Self-reported Health; MHI, Median Household Income; CBSA, Census Based Statistical Area; ZCTA, Zip Code Tabulation Area; PCA, Principal Component Analysis; SD, Standard Deviation, ACS, American Community Survey; Prop., proportion. Less common acronyms are defined on the table.

### Risk of bias

We assessed all 36 studies for potential bias that could have impacted their findings. Both qualitative and quantitative studies were assessed against 14 metrics of high-quality studies [13, 14]. In general, studies met most criteria and were deemed of relatively high quality with a low risk of bias. All studies explicitly stated the study question, adequately defined the study population, exposure, outcomes, and covariates. However, under the metrics used to indicate higher quality, many studies did not measure the exposure across more than one time period, instead using a binary definition to assess gentrification. This creates the potential to misclassify neighborhoods that gentrified, as neighborhoods not meeting thresholds for gentrification may still be changing enough to impact health. Despite not meeting this one metric, included studies met most of others, thus carrying an overall low risk of bias.

### Conceptualizing gentrification

The reviewed articles used multiple phenotypes to define gentrification. Among studies using a qualitative approach, gentrification was defined through investigator-determined changes in demographics, rent prices, or household income [17, 31, 33, 34, 40, 42, 43, 46, 47]. Studies that used a quantitative approach to define gentrification generally applied two major criteria [5, 16, 18–27, 29, 30, 32, 36–39, 41, 44, 45, 48–50]. First, neighborhoods had to be eligible to be gentrified. Eligibility was defined by comparing a neighborhood (e.g., census tract, block group) with a larger geographic area (e.g., city). Values for these variables generally had to be at or below the median of the larger geographic area or have a negative z-score compared to the larger geographic area. Second, there had to be a change in z-score value, percent, or numerical change from a baseline period to follow-up for a variable(s) of interest. Although most studies defined gentrification as a binary concept occurring over 2 time points, two studies described gentrification as a staged process [19, 23], using quintiles of z-scores or percentiles as indicators of different levels of gentrification.

Within these two parameters, the variables and number of variables used to define gentrification differed, as presented in Table 2. Only two studies used a single variable, one based on changes in educational attainment [45] and other based on changes to the white population [39]. Most studies used between 2 to 6 variables to define gentrification.

**Table 2:** Variables used to define and measure gentrification for included quantitative articles.

**Variable**	**N (%)**	**References**
Age (population)	5 (20.8%)	5, 16, 18, 29, 41
Age (housing stock)	3 (12.5%)	5, 24, 25
Education	17 (70.8%)	5, 16, 18, 19, 20, 21, 22, 23, 24, 25, 26, 27, 32, 38, 41, 45, 49
Home value	6 (25%)	5, 16, 20, 32, 38, 41
Median Household income	17 (70.8%)	16, 18, 19, 20, 21, 22, 23, 24, 25, 26, 27, 30, 32, 36, 38, 41, 49
Immigrant population (%)	2 (8.3%)	5, 41
Non-family households (%)	1 (4.2%)	16
Occupation	2 (8.3%)	16, 18
Poverty	4 (16.7%)	16, 23, 27, 38
Race	6 (25%)	16, 18, 22, 27, 38, 39
Rent	12 (50%)	16, 18, 20, 21, 34, 25, 26, 27, 30, 32, 38, 29
Urbanization	2 (8.3%)	24, 25
Owner Occupied	1 (4.2%)	16
Unemployment	1 (4.2%)	5
Residential Mobility	2 (8.3%)	5, 29
Dollar Amount of Improvement Loans (Per Capita)	1 (4.2%)	38
Mean Dollar Amount of Home Loans	1 (4.2%)	38
Composite Measure (SEIFA, IMD, and NCGS)	3 (12.5%)	37, 44, 48
**Geographic Level**	**N (%)**	**References**
Zip code	2 (8.3%)	18, 27
Census Tract	10 (41.7%)	5, 19, 20, 21, 22, 24, 25, 32, 45, 49
Block group	1 (4.2%)	16
Other	7 (29.1%)	23, 26, 29, 30, 41, 44, 48
**Time between baseline and follow-up (years)**	**N (%)**	**References**
5	2 (8.3%)	5, 44
10	14 (58.3%)	16, 18, 20, 21, 22, 24, 25, 26, 32, 36, 38, 39, 45, 49
20	3 (12.5%)	23, 27, 39
Other	5 (20.8%)	41, 19, 48, 29, 37
**Analytical Method**	**N (%)**	**References**
Logistic Regression Models	12 (50%)	18, 19, 20, 22, 24, 25, 26, 27, 29, 30, 37, 44
Linear Regression Models	8 (33.3%)	19, 21, 30, 32, 39, 45, 48, 49
Principal Component Analysis	2 (8.3%)	16, 26
Other	8 (33.3%)	5, 16, 23, 32, 36, 37, 38, 41

Across these studies, common variables incorporated in defining gentrification included neighborhood socioeconomic factors (e.g., median income, proportion with bachelor’s degree), measures of housing value and stock (e.g., median home value, proportion of housing greater than 20 years old, median rent price), and changing demographics (e.g., proportion of the population Black or White, proportion aged 30–44 years, or proportion aged 65+). Additional variables included urbanization of neighborhoods, change in immigrant population, and change in type of professions. Three studies used a composite index to define gentrifying areas. The indices, the Socioeconomic Index for Areas of Relative Advantage and Disadvantage (SEIFA) in Australia, the Index of Multiple Deprivation (IMD) in the United Kingdom, and the Neighborhood Change and Gentrification Scale (NCGS) in the United States, included a variety of variables that measure household income, employment, housing, occupation, education, material objects, and health.

Across studies, neighborhoods were also defined at different geographic levels, including zip code, census tract, and, infrequently, block groups. Investigators generally chose to conduct studies in geographic areas that were actively gentrifying or had already gentrified, including: Philadelphia [20, 22], Chicago [17, 29], New York City [18, 19, 23, 26, 30, 31, 33, 37, 40], Montreal [42, 49], Vancouver [43, 46], San Francisco [34], Baltimore [37], Melbourne [47], and Portland [39]. Several studies examined multiple geographic areas [21, 24, 25, 27, 37, 38, 48]. Studies conducted outside of the United States used different geographic areas, such as districts and census sections [41, 44, 45].

The time period most often used to quantify or describe the effects of gentrification was about 10 years [16, 18, 20–22, 24–26, 32, 36, 38, 39, 45, 49] with some studies using as few as 5 years [5, 44] and others up to 20 years [23, 27, 30].

### Health-related outcomes

#### Self-reported health

Six studies in this review examined the relationship between gentrification and self-reported health [21, 22, 25, 32, 36, 49]. Multiple reported that gentrification was not associated with or only marginally associated with higher self-reported health [21, 22, 25, 36, 49]. However, negative associations were found in analyses that examined specific subgroups, such as African Americans [22, 25] or higher-income older adults [32], with gentrification being associated with worse self-reported health.

#### Physical and mental health diagnoses

Six studies quantified the association between gentrification and biomarkers or disease symptoms/diagnoses [5, 19, 29, 32, 37, 38]. In one study, greater neighborhood affluence and gentrification was associated with a lower risk of hypertension [29]. Another study examining diabetes incidence found that compared to those living in “stable” areas, those living in “declining SES,” “new housing” and “improving SES” areas have a decrease in diabetes incidence [5]. One study considered a range of health measures in evaluating the relationship between gentrification and the health of low-income children, including BMI, asthma, attention-deficit/hyperactivity disorder (ADHD), conduct disorder, and anxiety or depression prevalence, emergency department visits and hospitalizations, and proportion of children with at least one well-child visit (or routine exam). Children in rapidly gentrifying areas experienced higher rates of anxiety and depression, though no relationship between gentrification and health was found for the other outcomes [19].

The impact of gentrification on mental health was also assessed by three other studies [32, 37, 38]. One found that compared to their counterparts in high income areas, both economically vulnerable and higher income older adults in gentrifying areas experienced more depression and anxiety symptoms [32]. The other found that low-income and long-term adults of gentrifying neighborhoods had a higher likelihood of serious psychological distress than those in low-income and not gentrified neighborhoods [38]. However, gentrification was not associated with the likelihood of having psychotic experiences [37].

#### Health related behaviors

Four studies examined the impact of gentrification on health-related behaviors and healthcare utilization [24, 26, 47, 48]. Two examined gentrification in relation to emergency department encounters. One found that displaced residents were more likely to have an emergency department encounter, experience hospitalizations, and make mental health visits as compared to residents who remained in gentrifying neighborhoods or non-gentrifying neighborhoods [26]. The other study found that decreases in the rate of emergency room admissions were not associated with gentrification [48].

Studies that examined the association between gentrification and alcohol use presented conflicting results. In one study, gentrification was not associated with increased drinking (i.e., binge drinking) overall but did increase the odds of binge drinking in residents who had lived in the neighborhood for less than 5 years [24]. Another study suggested that gentrifying areas are more likely to criminalize those who participated in public drinking, with several study participants reporting greater levels of isolation and increased drinking as a result [47].

#### Environmental changes that have implications for health or health behaviors

Seven studies considered the relationship between gentrification and environmental changes that impact health [16, 18, 30, 34, 39, 41, 45]. Four of these studies suggested that gentrification is related to changes in access to green space, air pollution, and walkability [16, 18, 41, 45]. Greater exposure to active green space was associated with lower odds of reporting fair or poor health [18]. However, within gentrifying neighborhoods, researchers found that the health benefits of increased green space only held for those with high incomes or high levels of education [18]. In another study, gentrification was found to occur in closer proximity to green spaces, though researchers suggested that this “green gentrification” may result in the displacement of socially vulnerable residents [41]. In regard to its impact on environmental hazards, one study found that air pollution levels are not equally distributed among neighborhoods that did and did not gentrify; neighborhoods that did not gentrify had a greater proportion of cumulative toxic air pollution compared to other neighborhoods [16].

Three studies examined the relationship between gentrification and food accessibility [30, 34, 39]. One study found that gentrification increases the prevalence of both healthy and (particularly) unhealthy food options [30] while one suggested that gentrification may increase food insecurity and force residents to spend a greater proportion of their income on housing, thereby leaving less to spend on nutritious food [34]. Another study examined the relationship between food mirages (areas with physical access to food but high food prices). It found that food mirages were more likely to be found in areas experiencing gentrification, suggesting that low-income individuals in these areas may not be able to benefit from increased access to food [39].

#### Moderators/Group differences

Several studies we reviewed emphasized that the impact of gentrification on a neighborhood is shaped by the demographics of the neighborhood; that is, gentrification may impact one population and not another [16–20, 22–25, 27, 32–34, 38, 43, 46, 47]. For example, Huynh and Maroko reported that although gentrification overall was not associated with preterm birth, very high gentrification—defined as neighborhoods with a substantial increase in college educated and high median household income—was adversely associated with preterm birth for non-Hispanic Blacks but protective of preterm birth in non-Hispanic Whites [23].

In another study, elevated self-reported stress levels, which have been shown to negatively impact health, were higher among non-White residents within neighborhoods that experienced White gentrification as compared to neighborhoods that had experienced Black gentrification [20]. Other studies also suggested that race may moderate the impact of gentrification on health, with Black individuals reporting worse self-reported health [22, 25].

Gentrification may also impact recovery efforts for drug users. Studies showed that injection drug users were more likely to be homeless in neighborhoods that gentrified [27] and were unable to access overdose prevention sites because of policing practices in gentrifying areas [43]. This can increase risks for multiple health conditions [51].

Studies suggested differential results on the impact of gentrification on health by age. Anxiety or depression diagnoses have been shown to be significantly greater among low-income children in rapidly gentrifying areas than those in persistently low SES areas, though gentrification was not associated with other health measures in this population [19]. In another study, gentrification was associated with increases in both healthy and unhealthy food options but was not associated with rates of childhood obesity [30]. Among older adults, gentrification inversely affected the self-reported health of those who were higher-income, while positively affecting that of those who were low-income, when compared to their counterparts in low-income neighborhoods [32]. Among older adults, gentrification was also attributed to a loss of social capital [33, 40, 42]

### Potential mechanisms

Our review also included studies that examined potential mechanisms mediating the association between gentrification and health. The current literature suggests that gentrification may impact health through social capital and psychosocial stress [17, 28, 31, 33, 35, 40, 42, 44, 46, 49].

#### Social capital

Social capital has been described as the collective value of all social networks and the inclinations that arise from these networks to do things for each other (norms of reciprocity) [52]. In the face of change, social capital can be critical in maintaining one’s health. One study examined this, finding that higher levels of identification or connectedness with one’s neighborhood can act as a buffer to protect individuals from mental health strains related gentrification [44].

In our literature search, five articles linked gentrification with a decline in necessary components of social capital: neighborhood trust, social cohesion, and/or social networks [17, 33, 40, 42, 46]. All these articles examined the impact of gentrification on vulnerable communities (e.g., the elderly and/or marginalized racial and sexual backgrounds). Versey and colleagues describe common themes among older Black residents of gentrifying neighborhoods. Participants reported a decrease in neighborhood trust, collective efficacy, and a disruption of social networks, outcomes related to poor health [33]. Another common narrative was perceived conflict between gentrifiers and long-term residents; the former of whom were seen as dismantling long standing community structures. Gentrification, though viewed positively by some, was generally attributed to a loss of social spaces for long-term residents [40, 42].

Only one study suggested that gentrification increases collective efficacy, another form of social capital [49]. Researchers hypothesized that incoming higher income residents, who may carry increased political influence and agency, could demand higher quality resources and promote community initiatives, benefitting long-term residents.

#### Psychosocial stress

Gentrification was also cited as a source of psychosocial stress [31]. Participants in several studies also noted shifting racial demographics accompanying gentrification, leading to perceived increases in preferences towards the White residents. Among non-white residents, this was seen as a source of elevated stress and decreased community cohesion [17, 31, 33, 40]. Gentrification was also cited as a cause of decreasing comfort for certain groups such sexual/gender minority identified sex workers [46] and people who use drugs [27, 43].

Research has attributed increased psychosocial stress to poor health outcomes [53]. Within the context of gentrification and social capital, one review suggested separation from a familiar environment and difficulty establishing new social networks may result in elevated plasma cortisol levels, more hospitalizations, and decreased overall wellbeing [28].

## Discussion

**Fig 2:**
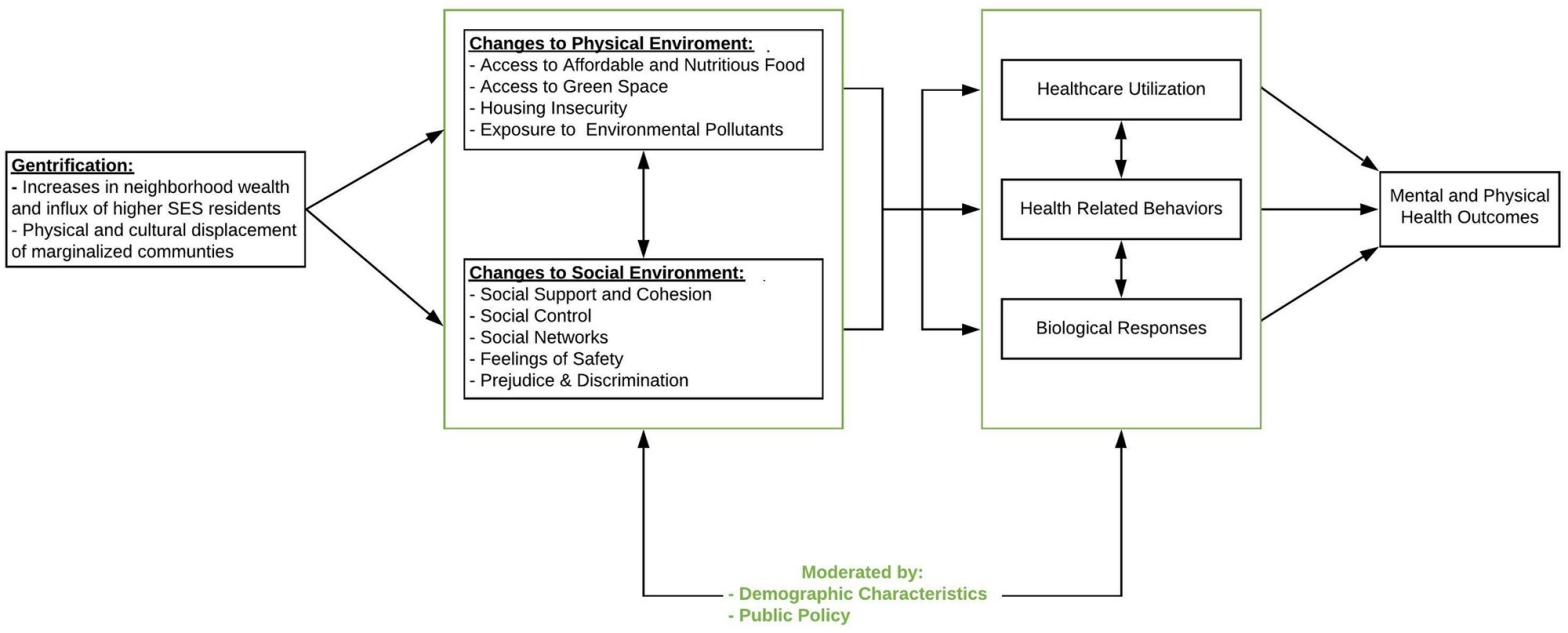
Conceptual model of gentrification and health.

Our systematic review of gentrification and health yields several insights that are valuable for future research in this area. Based on our review, we propose a conceptual model (Fig 2) that describes the process of gentrification as involving changes to both the physical and social environment, which in turn can indirectly affect health outcomes through several mediators. Based on accumulated evidence, we also suggest that characteristics like race and age may modify the association between gentrification and health, such that Black residents and the elderly may be more likely to have their health negatively impacted by gentrification than White and younger residents.

Our findings on the relationship between gentrification and health are consistent with prior research on the health impact of physical and social environments. In this context, the physical environment refers to the availability and quality of resources within a neighborhood, including transportation, access to healthy food, noise level, air pollution, and recreational resources. These factors impact behaviors such as physical activity, diet, and sleep. These behaviors, in turn, can affect proximal biological factors such as blood pressure, diabetes, and stress levels, which can increase risk for distal health outcomes such as cardiovascular disease [54, 55]. The social environment includes safety and violence, social support and cohesion, and social norms. These factors can impact psychosocial stress levels, which in turn affect proximal biological factors and thereby impact the risk of distal health outcomes [56, 57]. Our review finds that gentrification, specifically, can impact neighborhoods and the health of residents through environmental factors, stress, and social capital.

However, research on gentrification and health is limited by inconsistencies in defining gentrification. The qualitative studies we reviewed all defined gentrification differently, often using distinct sources to support their definitions. Among the quantitative studies, we identified common approaches in developing a definition. These approaches include 1) identifying neighborhoods that were eligible to be gentrified and 2) measuring a change in z-score value, percent, or numerical change from a baseline period to follow-up for a variable (s) of interest. However, within these two parameters, the number of variables used to define gentrification was inconsistent, varying from one to six. Based on the most central variables that have been used in the gentrification literature, we propose defining and quantifying gentrification based on changes to neighborhood socioeconomic status (median income, median home value/rent, educational attainment) and changing demographics (race and age).

In addition, most phenotypes of gentrification compared socioeconomic variables at the census tract or block group to a city-wide average. Implicitly, this suggests that gentrification is possible in any city, including places like San Francisco, Palo Alto, or even Beverly Hills. However, it is clear that these cities are not technically gentrifying, even if certain areas within them are below the city-wide mean, as they are already starting with strong labor markets, high employment, and other indicators of a high socioeconomic level. As such, novel phenotypes of gentrification are needed that do not require a comparison to the surrounding area. Lastly, gentrification is not a binary exposure, as it was defined by most studies; it is a process that can occur over a period of years and even decades. As such, the lack of granular socioeconomic data from prior decades at the same geographical level to define gentrification further impedes study of this phenomenon.

The limited number of health outcomes examined in gentrification literature presents another limitation to current literature. Linking neighborhood level data that defines gentrification with other sources of health data—such as the electronic health record (EHR)—may allow for a more robust examination of the association between gentrification and health [58]. Such approaches can also enable researchers to examine the health outcomes of those who may have been displaced by gentrification, another challenge faced by most of the reviewed studies.

By linking multiple data sources and collecting primary data—such as biomarkers—we might also be able to address a third gap in knowledge: the paucity of research on the mechanism(s) by which gentrification may impact health. One possible mechanism by which gentrification impacts health is through social capital. Prior work has shown that strong levels of social capital and its related constructs, such as collective efficacy, social networks, community cohesion, and social fabric are associated with multiple positive health outcomes, including: lower obesity prevalence [59], fewer hospitalizations from coronary heart disease [60], lower prevalence of sexually transmitted diseases [61], higher levels of self-reported health [62], and decreased mortality [63].

However, the way in which gentrification affects social capital remains contested. Some have proposed that gentrification results in greater social capital, providing disadvantaged communities with increased opportunities to access individuals and institutions associated with mainstream society [64]. Research also suggests that gentrification erodes social capital, particularly for disadvantaged groups [8–10]. Policies that promote gentrification and a decrease in social cohesion may lead to the displacement [65, 66] and socio-spatial segregation [11] of cities, exacerbating social capital. Further research on the mechanisms by which gentrification may impact health is critical and necessary in answering debates like this one.

### Limitations

All effort was made to ensure that a comprehensive literature search was conducted. It is possible that articles were missed for various reasons, including for not being published in English or gentrification being described using different terminology. Additionally, there is the possibility of publication bias, but we did identify articles showing no association between gentrification and health in our review.

A novel conceptualization of the association between gentrification and health is needed to inform this future research, including mechanistic studies and interventions that can mitigate any negative impact of gentrification. Fig 2 depicts our conceptualization, based on current literature, of how gentrification is associated with health. Much like prior models that depict how neighborhoods impact health, we propose gentrification changes the physical and social environment, which in turn can result in biological responses (e.g., increased levels of stress), changes in health-related behaviors (e.g., physical activity, management of pre-existing conditions), and/or changes in healthcare utilization (e.g., emergency department visits, preventative care). These proximal impacts may increase or decrease risk for long term mental and physical health outcomes. All of these changes occur over time and can be moderated by public policy such as economic incentives and subsidized housing.

## Conclusion

Gaps in knowledge inform future directions for this line of research. Developing a consensus definition of gentrification requires novel phenotypes of gentrification, specifically ones that do not rely on relative comparisons to surrounding geographies. Based on the current literature, a core set of health outcomes that should be included in studies that examine the association between gentrification and health include measures of mental and physical health (e.g., depression, anxiety, hypertension, diabetes) and healthcare utilization (e.g., primary care encounters). There is a need for more proximal measures of health—such as biomarkers—since gentrification may not impact incidence of hard outcomes (e.g., myocardial infarction, stroke) in the short term. All of this should be done using analytic approaches that incorporate other data sources, including information on the pollutants in the environment and the physical resources available to residents. The results of these ongoing questions may inform interventions to address this social determinant of health.

## Supporting information

S1 Table 2009 PRISMA checklist. (DOC)

S1 Appendix Final search strings used in review. (DOCX)
