# Supplementary material for: Defining gentrification for epidemiologic research: A systematic review
Source: PLoS One. 2020 May 21;15(5):e0233361. doi: 10.1371/journal.pone.0233361 (PMC7241805; doi:10.1371/journal.pone.0233361)
Supplement: S1 Appendix — (DOCX) [file pone.0233361.s002.docx]

|  | Gentrification | Health | Mechanisms/Moderators by Which Gentrification may Affect Health |
| --- | --- | --- | --- |
| **Pubmed (Initial)** | gentrification |  |  |
| **Web of Science** | gentrification displacement relocation  urbanization urbanisation urban renewal  housing eviction | health social determinant | inequity equity disparity discrimination segregation unequal insecurity poverty disadvantage  socioeconomic health Status Disparities social capital vulnerability |
| **Pubmed (second)** | gentrification displacement relocation  urbanization urbanisation urban renewal  housing eviction | health social determinant | inequity equity disparity discrimination segregation unequal insecurity poverty disadvantage  socioeconomic health Status Disparities social capital vulnerability race minority  racial ethnicity minority Groups |

**PubMed Search String**

*Initial Searches:*

gentrification

*Second:*

((((gentrif*[tiab] or displac*[tiab] or relocat*[tiab] or urbanization[tiab] or urbanisation[tiab] or “urban renewal”[tiab] or evict*[tiab])) AND (Health[tiab] or "social deter*"[Mesh])) AND (race[tiab] or minorit*[tiab] or racial[tiab] or ethnic*[tiab] or "Minority Groups"[Mesh])) AND (inequ*[tiab] or equit*[tiab] or dispar*[tiab] or discrim*[tiab] or segreg*[tiab] or unequal*[tiab] or insecur*[tiab] or poverty[tiab] or disadvantag*[tiab] or socioeconomic*[tiab] or "Health Status Disparities"[Mesh] or “social capital”[tiab] or “vulnerab*”[tiab])

**Web of Science**

TOPIC: (gentrif* or displac* or relocat* or evict*) *AND* TOPIC: (health or “social deter*”) *AND* TOPIC: (inequ* or equit* or dispar* or discrim* or segreg* or unequal* or insecur* or poverty or disadvantag* or socioeconomic* or "social capital" or vulnerab*)

Timespan: All years. Indexes: SCI-EXPANDED, SSCI, A&HCI, CPCI-S, CPCI-SSH, BKCI-S, BKCI-SSH, ESCI, CCR-EXPANDED, IC.
